# An improved chromosome-level genome resource for the sheep scab mite, *Psoroptes ovis*

**DOI:** 10.1128/mra.00039-26

**Published:** 2026-08-04

**Authors:** Eilidh Geddes, Fernando Cruz, Jessica Gomez-Garrido, Marta Gut, Wouter van Mol, Roel Meyermans, Kathryn Bartley, Sara Roose, Jack Hearn, Stephane Rombauts, Steven Janssens, Yves Van de Peer, Peter Geldhof, Edwin Claerebout, Nadine Buys, Ivo G. Gut, Tyler Alioto, Stewart T. G. Burgess

**Affiliations:** 1 Moredun Research Institute, Edinburgh, Scotland, United Kingdom; 2 Centro Nacional de Análisis Genómico (CNAG), Barcelona, Spain; 3 Universitat de Barcelona (UB) 16724, Barcelona, Spain; 4 Laboratory of Parasitology, Faculty of Veterinary Medicine, Ghent University 86797, Merelbeke, Ghent, Belgium; 5 Department of Biosystems, Center for Animal Breeding and Genetics, KU Leuven 26657, Leuven, Belgium; 6 Centre for Epidemiology and Planetary Health, School of Veterinary Medicine and Biosciences, SRUC 735917 , Inverness, United Kingdom; 7 Department of Plant Biotechnology and Bioinformatics, Ghent University 26656, Ghent, Belgium; 8 VIB Centre for Plant Systems Biology, Ghent, Belgium; University of California Riverside, Riverside, California, USA

**Keywords:** *Psoroptes ovis*, genomes, sheep scab

## Abstract

Sheep scab, caused by infestation with the ectoparasitic mite, *Psoroptes ovis*, represents a major welfare and economic challenge for the livestock industry. We present an updated 62.7 Mb genome assembly containing 10 chromosome-level scaffolds with 10,516 annotated protein-coding genes, which provides a useful resource for studies into resistance and control.

## ANNOUNCEMENT

*Psoroptes ovis*, the causative agent of the ectoparasitic disease psoroptic mange (sheep scab), is a major welfare concern to the global livestock industry, costing the UK industry £80–200 M/annum (1, 2). Host-adapted *P. ovis* can also infest other species, for example, cattle and rabbits (3, 4). Infestation is spread through direct contact or fomites and causes intense pruritus, secondary bacterial infections, emaciation, and potentially death (5). Recent evidence of *P. ovis* resistance to the macrocyclic lactones, one of two licensed treatments (6, 7), presents significant threats to control, which this improved genome assembly can address.

A mixed population of sheep-derived *P. ovis* mites was obtained from a lab isolate cultured *in vivo* at Moredun Research Institute, Edinburgh, for approximately 25 years (ethical approval ref: E03/23).

After washing with 0.01% Triton X-100 and grinding mites under liquid nitrogen, high-molecular-weight DNA was extracted using SDS-proteinase K (8). Oxford Nanopore Technologies (ONT) long-read sequencing libraries were prepared using ligation 1D sequencing SQK-LSK109 and run on a GridION Mk1 for 72 h using a R9.4.1 flow cell (FLO-MIN106). gDNA underwent end-repair and adenylation using the NEBNext UltraII End Repair/dA-Tailing Module, with ligation of adaptors and purification using 0.4× AMPure XP Beads. Sequencing quality was monitored with MinKNOW v4.2.5 and basecalling performed using Guppy v4.3.4 with the dna_r9.4.1_450bps_modbases_dam-dcm-cpg_hac model. Length and quality filtering was performed with Fitlong v0.2.0 with a target yield of 4.0 Gb. Preparation of 10× library was performed using the Chromium Controller instrument and Genome Reagent Kits v2 (10× Genomics) following manufacturer’s protocols. Illumina-compatible paired-end sequencing libraries were prepared and sequenced on Illumina NovaSeq 6000 (2 × 151 bp), following manufacturer’s recommendations.

**Fig 1:**
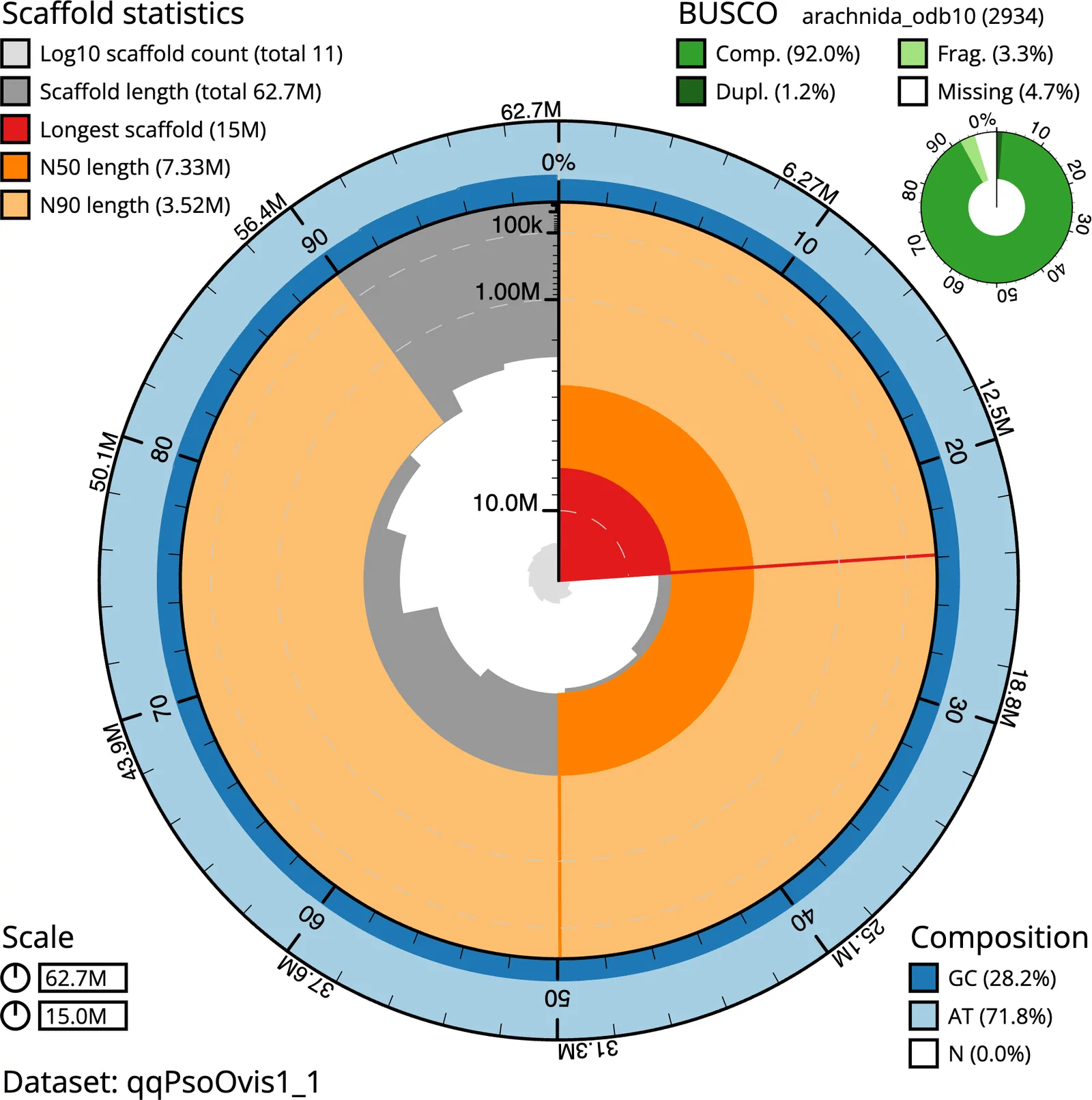
Snail plot summary of assembly. The main plot is divided into 1,000 size-ordered bins around the circumference, with each bin representing 0.1% of the 62.7 Mb assembly. The distribution of sequence length is shown in dark gray, with the plot radius scaled to the longest sequence (15 Mb, in red). Orange and pale-orange arcs show scaffold N50/N90 sequence lengths (7.33 Mb/3.53 Mb), respectively. Pale gray spiral shows cumulative sequence count on log-scale, with white scale lines showing successive orders of magnitude. Blue and pale-blue area around outside of the plot shows distribution of GC, AT, and *N* percentages in the same bins as the inner plot. A summary of complete, fragmented, duplicated, and missing BUSCO genes from the arachnida database (odb10) is shown in the top right.

Genome assembly was carried out using CLAWS v1.0 (https://github.com/cnag-aat/CLAWS). Briefly, the ONT data were assembled with Flye (v2.8.3, [9]) and scaffolded with 10× data, resulting in two joins. The assembly contains 13 contigs in 10 large scaffolds and 1 small unplaced scaffold, with a total assembly span of 62.7 Mb (Table 1, Fig. 1), with greater continuity than the previous assembly (contigs = 93 [10]).

**TABLE 1:** *Psoroptes ovis* genome sequencing, assembly, and annotation statistics

Metric	Statistic
Sequencing	
ONT reads (raw)	2,775,079
ONT yield (raw)	21.5 Gb (343×)
ONT read N50 (raw)	13 kb
ONT reads (filtered)	133,570
ONT yield (filtered)	4.00 Gb (64×)
ONT read N50 (filtered)	32 kb
10× Genomics (2 × 151 PE Illumina) yield	62.7 Gb (1,000×)
10× Genomics read pairs	207,565,000
Assembly	
Span	62,663,122
Contig N50	7,334,676
Scaffold N50	7,334,676
Number of contigs	13
Number of scaffolds	11 (10 autosomes plus mtDNA)
Number of gaps	2
Consensus quality (QV)	43.3
BUSCO score	C: 92.0% [S: 90.8%, D: 1.2%], F: 3.3%, M: 4.7%, *n*: 2,934 (v5.5.0)
Annotation	
Number of protein-coding genes	10,516
Median gene length (bp)	1,903
Number of transcripts	14,782
Number of exons	46,576
Number of coding exons	42,137
Median UTR length (bp)	404
Median intron length (bp)	75
Exons/transcript	4.27
Transcripts/gene	1.4
Multi-exonic transcripts	0.87
Gene density (genes/Mb)	167.8

Gene annotation was obtained by combining transcript and protein alignments and *ab initio* gene predictions. RNA-sequencing (RNA-seq) reads from the same *P. ovis* isolate National Center for Biotechnology Information (NCBI) (PRJNA521406) (11) were aligned with STAR (12) v2.7.2a. Transcript models were generated using Stringtie (13) v2.1.4 and combined using TACO (14) v0.6.3. Proteomes from related mites were aligned to the genome (Spaln2 [15]): *Tetranychus urticae*, *Sarcoptes scabiei*, *Euroglyphus maynei*, *Dermatophagoides pteronyssinus*, and *Varroa destructor* (UP000015104, UP000070412, UP000194236, UP000515146, and UP000594260, respectively). *Ab initio* gene predictions were performed with GeneID (16) v1.4, Augustus (17) v3.3.4, and Genemark-ES (18) v2.3e with and without RNA-seq data and data combined into consensus coding sequence models using EvidenceModeler-1.1.1 (19).

Functional annotation was performed with Blast2go (20) using Diamond Blastp (21) against NCBI nr database (January 2022) Interproscan (22) to detect protein domains. Non-coding RNAs were annotated using cmsearch (23) v1.1 against the FRAM database (24) v12.0.

In total, 10,516 protein-coding genes producing 14,782 transcripts (1.4 transcripts/gene) were annotated, encoding 13,361 unique proteins, and putative functions were assigned to 61.62% of annotated proteins (*n* = 8,233) (Table 1).

## Data Availability

All sequencing data for this project have been deposited in the International Nucleotide Sequence Database Collaboration (INSDC) databases under the umbrella project PRJEB82899, which contains the following two BioProjects: PRJEB82890 (read data) and PRJEB110042 (genome assembly). The ONT read data (ERX13401841) and 10× linked reads (ERX13401842) are included in study PRJEB82890. The annotated genome assembly (GCA_982319595.1) has been deposited in the study PRJEB110042.
